# Zinc improves learning and memory abilities of fetal growth restriction rats and promotes trophoblast cell invasion and migration via enhancing STAT3-MMP-2/9 axis activity

**DOI:** 10.18632/oncotarget.23122

**Published:** 2017-12-09

**Authors:** Lu Zong, Xiaohua Wei, Wenli Gou, Pu Huang, Ye Lv

**Affiliations:** ^1^ Department of Gynaecology and Obstetrics, The First Affiliated Hospital of Xi'an Jiaotong University, Xi'an, Shaanxi 710061, P.R. China; ^2^ Department of Behavioral Pediatrics, The Second Affiliated Hospital of Xi'an Jiaotong University, Xi'an, Shaanxi 710004, P.R. China

**Keywords:** fetal growth restriction, learning and memory abilities, zinc, trophoblast, matrix metalloproteinase-2/9

## Abstract

Fetal growth restriction (FGR) is a well-known risk factor for cognitive dysfunction, especially for learning and memory abilities. However, knowledge about prevention and treatment methods of learning and memory abilities of fetal are limit. Here, Morris water maze and passive avoidance tests showed zinc supplementation could protect the impairment of the learning and memory abilities caused by FGR. As accumulating evidence suggested that insufficiency of placental trophoblast cell invasion was closely related to FGR fetal neurodevelopmental dysplasia, we further explored the relationship between zinc supplementation during pregnancy and placental trophoblast. Microarray identified 346 differently expressed genes in placental tissues with and without zinc supplementation, and GO and KEGG analyses showed these differently expressed genes were highly enriched in cell invasion and migration and STAT3 pathway. Protein-protein interaction(PPI) analysis found that STAT3 interacted with matrix metalloproteinase-2/9 (MMP-2/9). *In vivo*, western blot results authenticated that the expression levels of phospho-STAT3, STAT3, MMP-2 and MMP-9 were up-regulated in placental tissues after zinc treatment. To validate whether zinc could promotes trophoblast cell invasion and migration via enhancing STAT3-MMP-2/9 activity. *In vitro*, Transwell assay was performed, and we observed that abilities of invasion and migration were obviously increased in zinc treated trophoblast cells. And phospho-STAT3, STAT3, MMP-2 and MMP-9 expression levels were correspondingly increased in zinc treated trophoblast cells, which were dose-dependent. Moreover, gain–of-function and loss-of-function of STAT3 confirmed that zinc promotes cell invasion and migration via regulating STAT3 mediated up-regulation of MMP-2/9 activity. We propose that activation of MMP-2/9 mediated by STAT3 may contribute to invasion and migration of trophoblast cells, which improved neurodevelopmental impairment of FGR rats probably via contributing to placental development. Our findings are the first to show a possible mechanism of reversing neurodevelopmental impairment of FGR rats by zinc supplementation, holding promise for the development of novel therapeutic modalities for learning and memory abilities impairment caused by FGR.

## INTRODUCTION

Fetal growth restriction (FGR) defined as estimated fetal weight below the 10th percentile, which could lead to a variety of diseases include neonatal encephalopathy, cerebral palsy, sepsis, seizures, respiratory distress, *etc* [1]. Thereinto, cognitive dysfunction is primary influencen on fetal [2], mainly manifested on learning and memory ability [3]. Children from pregnancies with FGR have demonstrated significantly lower academic achievement in school as well as lower professional achievement in adulthood [1]. It’s vital to avoid neurodevelopment impairment caused by FGR.

Fetal growth is a complex process involving maternal, placental and fetal factors [4]. More and more research confirmed that placental insufficiency is the principal cause of neurodevelopment impairment caused by FGR, resulting in chronic fetal hypoxia. This hypoxia induces a fetal adaptive response of cardiac output redistribution to vital organs, including the brain [2]. Proper placental development is essential for fetal growth and neurodevelopment, which need assistance from extravillous trophoblast(EVT) [1]. These cells migrate and invade into the uterine wall, leading to remodeling of the maternal vasculature [1, 5]. This remodeling yields a low-resistance, high-capacity perfusion system that allows for adequate exchange of oxygen, nutrients and key molecules at the maternal-fetal interface [1, 6]. However, insufficient trophoblast invasion has been associated with neurodevelopmental impairment caused by FGR [7, 8]. Therefore, improving placental development may be a key way to reverse it. However, knowledge about prevention methods about neurodevelopmental impairment caused by FGR was limit.

Micronutrients is one of the major determinants of pregnancy outcome, and play a key role in the development and functional maturation of the brain and central nervous system and also regulate the levels of neurotrophic factors [9]. For example, our previous study confirmed that vitamin D could increase the learning and memory ability of FGR rats, significantly ameliorating the cognitive dysfunction of FGR rats [3]. Recently, study found low placental zinc concentration induces fetal growth restriction during pregnancy [10], and the important role of zinc in neuroprotection has been observed in other study, which affects neurotransmitter receptors and synaptic plasticity [11]. However, whether zinc supplementation could directly reverse learning and memory abilities of fetal caused by FGR was unknown.

In this report, we investigate a novel mechanism which zinc improves learning and memory abilities of FGR rats by increasing migration and invasion ability of trophoblast cells. We find that zinc supplementation improved the learning and memory ability of FGR rats. We provide evidence that zinc supplementation increased expression of MMP-2/9 via enhancing transcription factor STAT3 activity, contributing to migration and invasion of EVT cells. The high expression of MMP-2/9 significantly contributed to cell invasion [12]. We propose that zinc supplementation may be a potential therapeutic strategy of learning and memory abilities impairment caused by FGR.

## RESULTS

### Zinc supplementation improves brain weight of FGR rats

**Figure 1:**
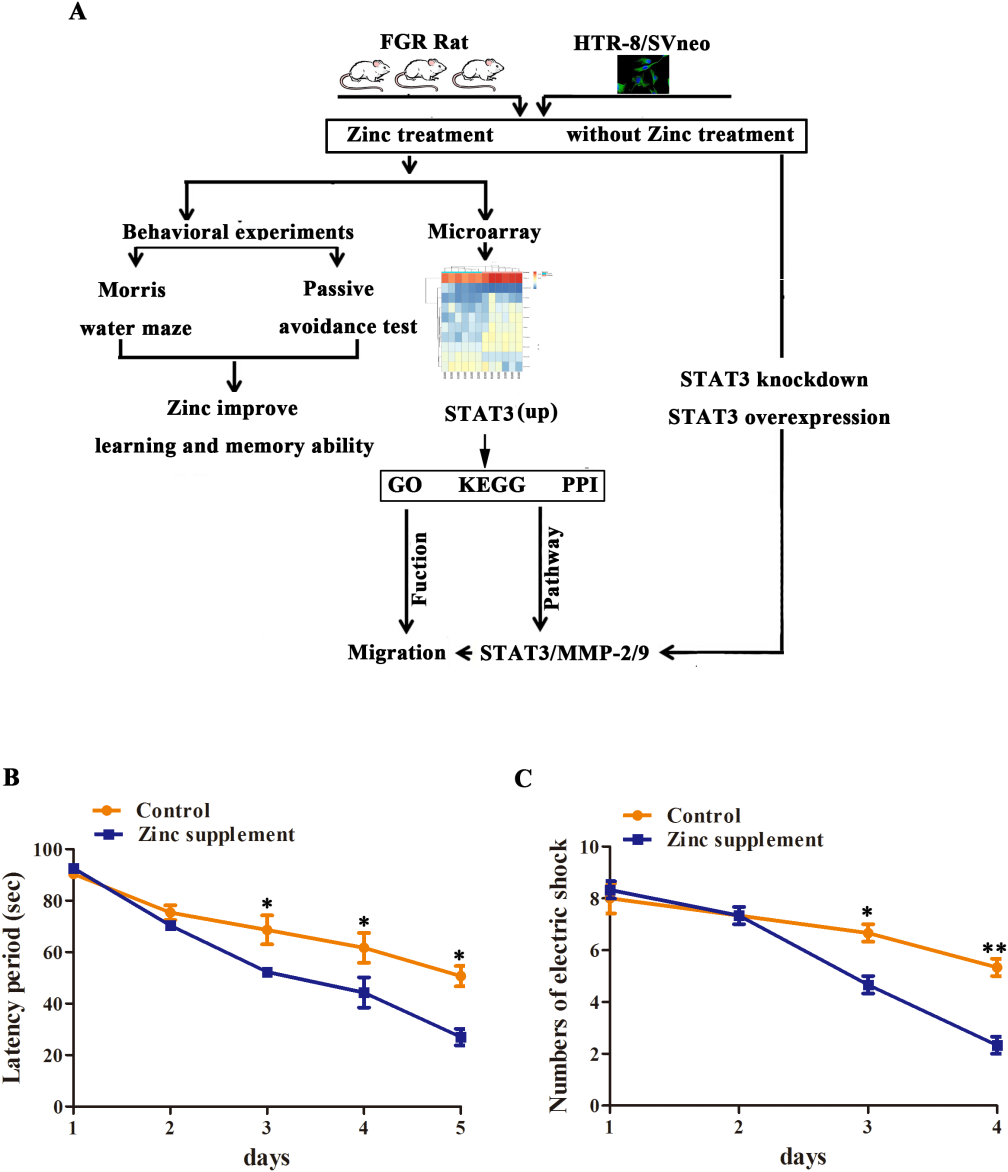
Flow chart of effects and molecular mechanism of Zinc on memory and learning abilities of FGR rats **(A)** Flow chart; **(B)** comparison of the results of the Morris water maze test between the two groups at different times; **(C)** comparison of the results of the passive avoidance test between the two groups at different times. ^*^: *P*<0.05; ^**^: *P*<0.01.

As shown in flowchart of experimental design (Figure 1A), pregnant rats were fed with high zinc diet. And the level of zinc was measured on 1, 7, 15 and 30 days, respectively. From 7 days with high zinc diet, we observed that the level of zinc was obviously increased in zinc supplementation group compared to control group(*P* < 0.01, Table 1). Accordingly, the brain weights of offspring rats aged 1, 7, 14 and 21 days in the Zinc supplementation group were significantly higher than those in the control group (*P* < 0.05), but no obvious difference was found in the comparison of the brain weights of rats aged 30 days between the two groups (*P* > 0.05, Table 2). In brief, zinc supplementation during pregnancy plays an important role for brain weight improvement of FGR rats.

**Table 1:** Comparison of the levels of zinc in two groups of rats (nmol/l)

Groups	Day 1	Day 7	Day 15	Day 30
Control (n = 20)	31.67±2.52	53.11±3.23	57.01±2.13	62.41±3.56
Zinc supplement (n = 20)	32.12±2.56	34.57±3.34	41.50±1.12	42.30±2.60
t	0.542	14.234	17.457	23.054
*P* value	0.657	0.0043	0.0012	0.0001

**Table 2:** Comparison of the weight of rat brain in two groups (g)

Groups	Day 1	Day 7	Day 14	Day 21	Day 30
Control(n = 20)	0.166±0.012	0.374±0.022	0.762±0.054	0.985±0.062	1.398±0.062
Zinc supplement(n = 20)	0.224±0.013	0.695±0.043	1.121±0.073	1.343±0.018	1.396±0.054
t	3.209	11.464	8.648	1.157	0.324
*P* value	0.004	<0.0001	0.001	0.045	0.865

### Zinc improves learning and memory abilities of FGR rats

To further the effect of zinc supplementation on learning and memory abilities of FGR rats, classical water maze and passive avoidance tests were performed [13]. For Morri water maze assay, a shortened trend was identified in the latency of each group after the rats were trained. In the first 2 days, there is no significant difference between zinc group and control group (*P* > 0.05). From the third day, the latency periods was shorter in zinc supplementation group that those in the control group (*P* = 0.013, 0.024 and 0.017, respectively; Figure 1B). In passive avoidance assay, with the training being carried out, the number of electric shocks that the rats from the two groups experienced was gradually decreased. On the third and fourth day, the numbers of electric shock in the zinc supplementation group were significantly less than those in the control group (*P* = 0.019 and *P* = 0.0037, respectively; Figure 1C). According to the above experiment results, we speculated that zinc supplementation during pregnancy improved learning and memory abilities of FGR rats.

### Zinc associates with placental cell invasion and migration

**Figure 2:**
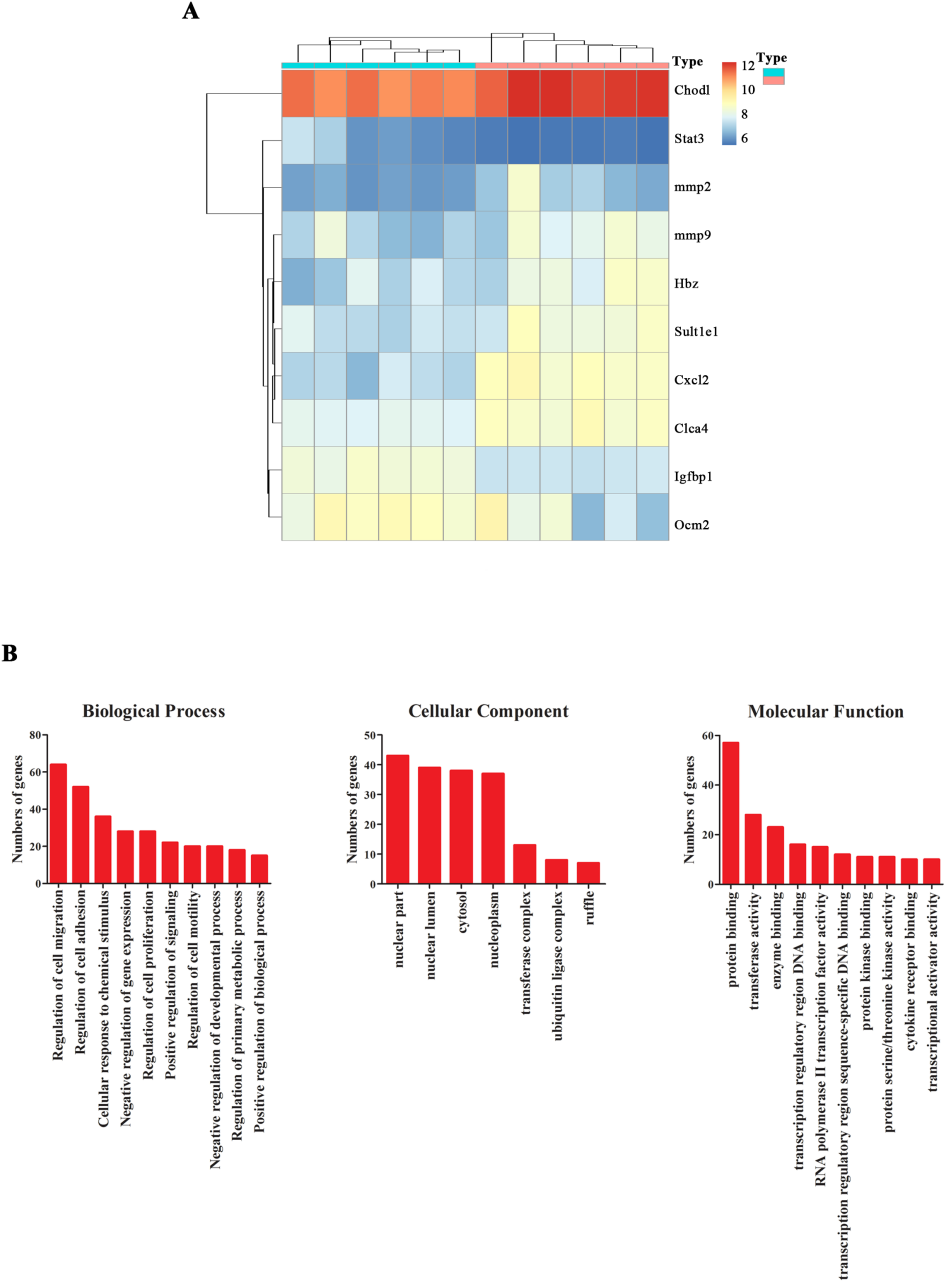
Differentially expressed genes and functions in placenta tissues with and without zinc supplementation **(A)** Heat map of differentially expressed mRNAs in placenta tissues with and without zinc supplementation(Blue stands for without zinc supplementation group; Red stands for with zinc supplementation group). **(B)** Gene ontology(GO) analysis of these differentially expressed genes.

More and more studies confirmed that neurodevelopment following fetal growth restriction is closely related to placental dysfunction [14]. To observe the effect of zinc supplementation on the placenta, mRNA microarray assay was performed to identify the novel differentially expressed mRNAs of placenta after Zinc treatment. Six pairs of pregnant rats placenta tissues from zinc supplementation and control groups were compared by mRNA microarray. Among all the detected mRNAs(28,000), 346 differentially expressed mRNAs (*P* < 0.01; 1< logFC< −1) were identified (Figure 2A). To further explore the functions of these differentially expressed mRNA, GO analysis was performed. Results revealing that these genes mainly involved in regulating primary metabolic, chemical stimulus, gene expression-, especially for migration had a very high ranking (Figure 2B).

### STAT3-MMP-2/9 pathway is obviously activated in placenta tissues after zinc supplementation

**Figure 3:**
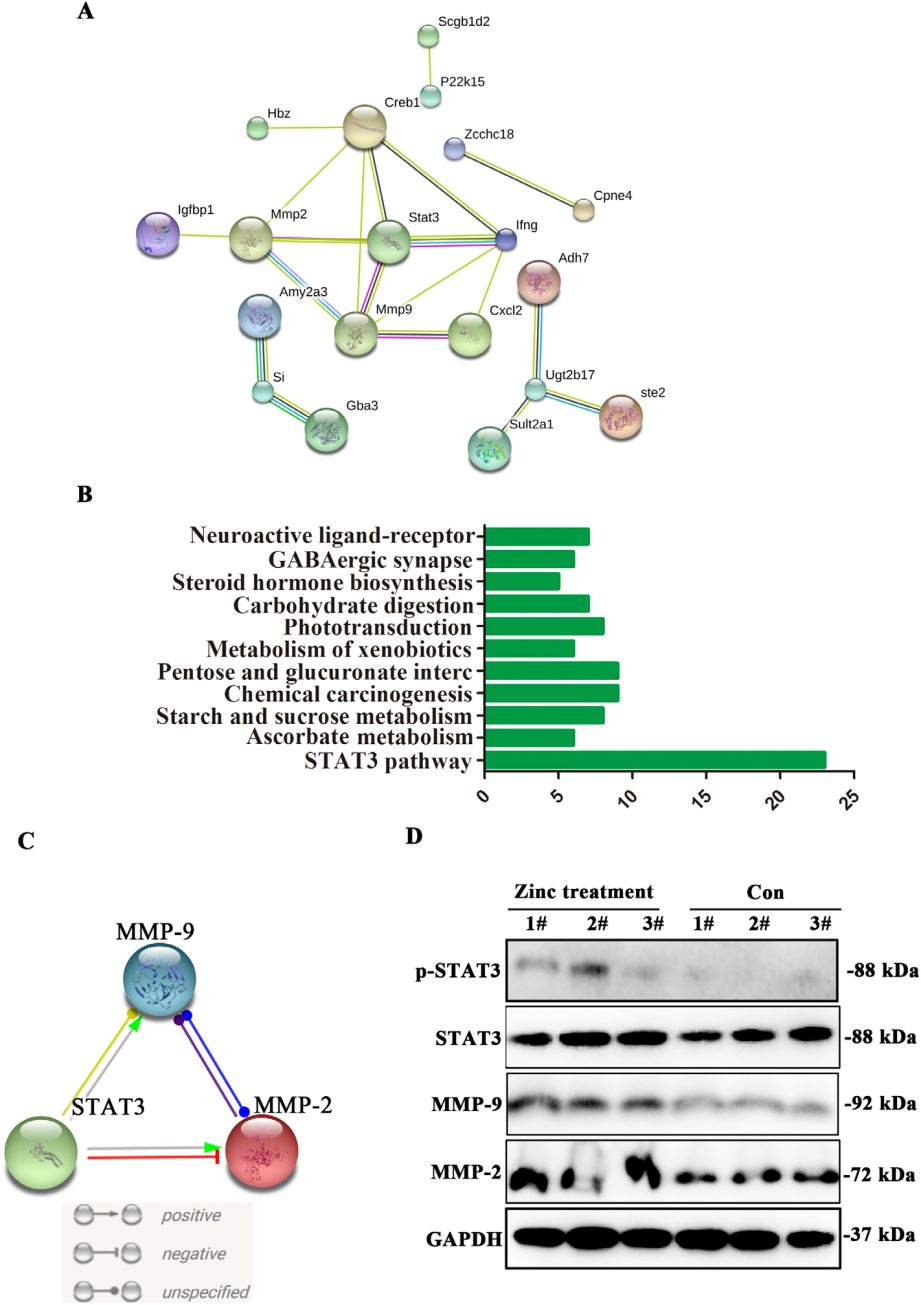
The expression level of STAT3-MMP-2/9 were upregulated after zinc supplementation **(A)** Protein-protein interaction of main dysregulated genes. **(B)** KEGG analysis of main dysregulated genes. **(C)** The interaction was existed among STAT3, MMP-2 and MMP-9. **(D)** The expression levels of STAT3, phosho-STAT3, MMP-2 and MMP-9 were obviously upregulated in placenta tissues after zinc supplementation.

Insufficient migration of trophoblast cell is the main reason of neurodevelopmental dysplasia of FGR fetal [1]. Hence, we further seek out the key molecular pathway related to cell migration after zinc supplementation. In migration gene sets, ten differentially expressed genes are shown in Table 3. To identify the key dysregulated genes in placenta tissues, PPI analysis was performed, and the results suggested that STAT3 was the core of protein-protein interaction network (Figure 3A). Consistent with PPI results, KEGG analysis confirmed that these differentially expressed genes mainly enriched in STAT3 signaling pathway (Figure 3B). To further explore the key axis mediated by STAT3, PPI analysis was performed again. Results revealing that MMP-2/9 are the important interaction genes with STAT3, and molecular action analysis also suggested that STAT3 positively associated with MMP-2/9 expression (Figure 3C). In order to verify above results, western blot was performed to detect phospho-STAT3, STAT3, MMP-2 and MMP-9 expression level in placenta tissues, and results suggested that phospho-STAT3, STAT3, MMP-2 and MMP-9 expression level was obviously up-regulated in placenta tissues treated by zinc compared to control group (Figure 3D). Above results strongly revealed that activation of STAT3/MMP-2/9 axis may be caused by zinc supplementation.

**Table 3:** Main dysregulated genes in zinc treatment and control group

No.	Gene name	logFC	t	*P* value
1	Chodl	−1.1219293	−13.667215	2.39E-09
2	Stat3	4.1215741	11.4243308	2.29E-08
3	mmp2	3.07057907	9.73855694	1.62E-07
4	mmp9	2.03463611	7.20885576	5.21E-06
5	Hbz	1.19483101	4.63714716	0.000410527
6	Sult1e1	−1.0256868	−3.484425	0.003774591
7	Cxcl2	1.13936286	3.15499728	0.007213986
8	Clca4	1.14957342	3.06978037	0.008529261
9	Igfbp1	1.05370532	2.60901567	0.020955273
10	Ocm2	−1.2291303	−2.5396303	0.023951714

### Zinc promotes migration and invasion of HTR-8/SVneo cells and up-regulates STAT3-MMP-2/9 expression

**Figure 4:**
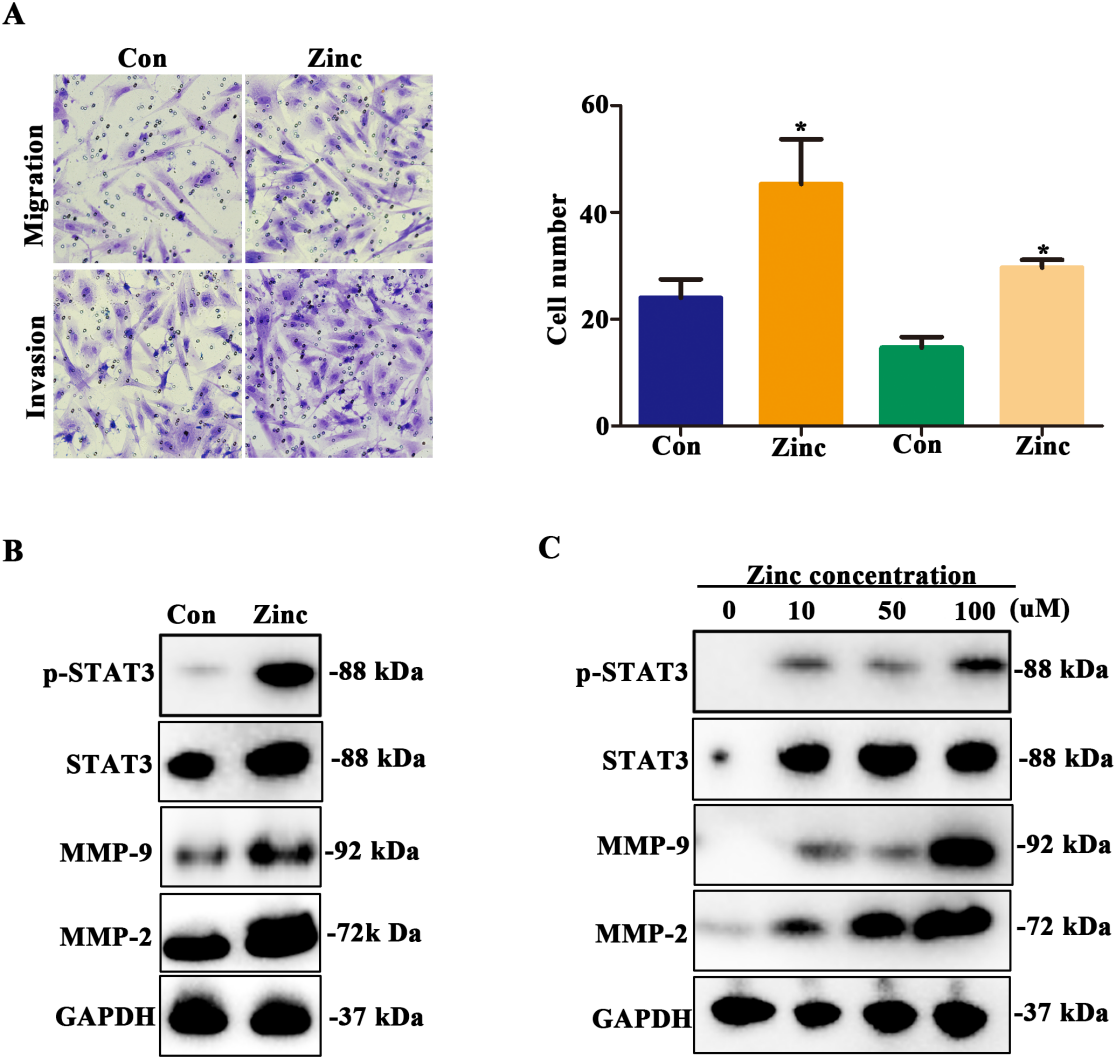
Zinc promotes HTR-8/SVneo cells migration and invasion and increases activity of MMP-2/9 **(A)** Transwell assay assessed the abilities of migration and invasion of HTR-8/SVneo cells with and without zinc treatment. **(B)** The expression levels of phospho-STAT3, STAT3, MMP-2 and MMP-9 in HTR-8/SVneo cells with and without zinc treatment. **(C)** The expression level of phospho-STAT3, STAT3, MMP-2 and MMP-9 in HTR-8/SVneo cells treated with different zinc concentrations.

It is generally known that MMP-2/9 promotes cell invasion and migration via STAT3 signaling pathway [15]. *In vitro*, we further explore whether zinc treatment could increase invasion and migration abilities and up-regulate STAT3-MMP-2/9 activity. In migration assay, we observed that HTR-8/SVneo cell numbers transfered membrane was higher in zinc treatment group compare to untreated group at 24 hours (Figure 4A, *P* < 0.05). For invasion assay, untreated HTR-8/SVneo cells had a low ability to migrate and penetrate a Matrigel matrix, contrasting with the high capacity of those cells to migrate and invade in the presence of zinc (Figure 4A, *P* < 0.05). Next, western blot assay was performed to detected the STAT3-MMP-2/9 expression level, and results suggested that the expression levels of phospho-STAT3, STAT3, MMP-2 and MMP-9 were significantly up-regulated in zinc treated HTR-8/SVneo cells (Figure 4B). Moreover, with the increase of zinc concentration, their expression levels were also increased accordingly (Figure 4C). In brief, we verified that zinc promotes migration and invasion of HTR-8/SVneo cells and up-regulates STAT3-MMP-2/9 expression, which was in a dose-dependent manner.

### Zinc promotes migration and invasion of HTR-8/SVneo cells via up-regulating MMP-2/9 expression mediated by STAT3

**Figure 5:**
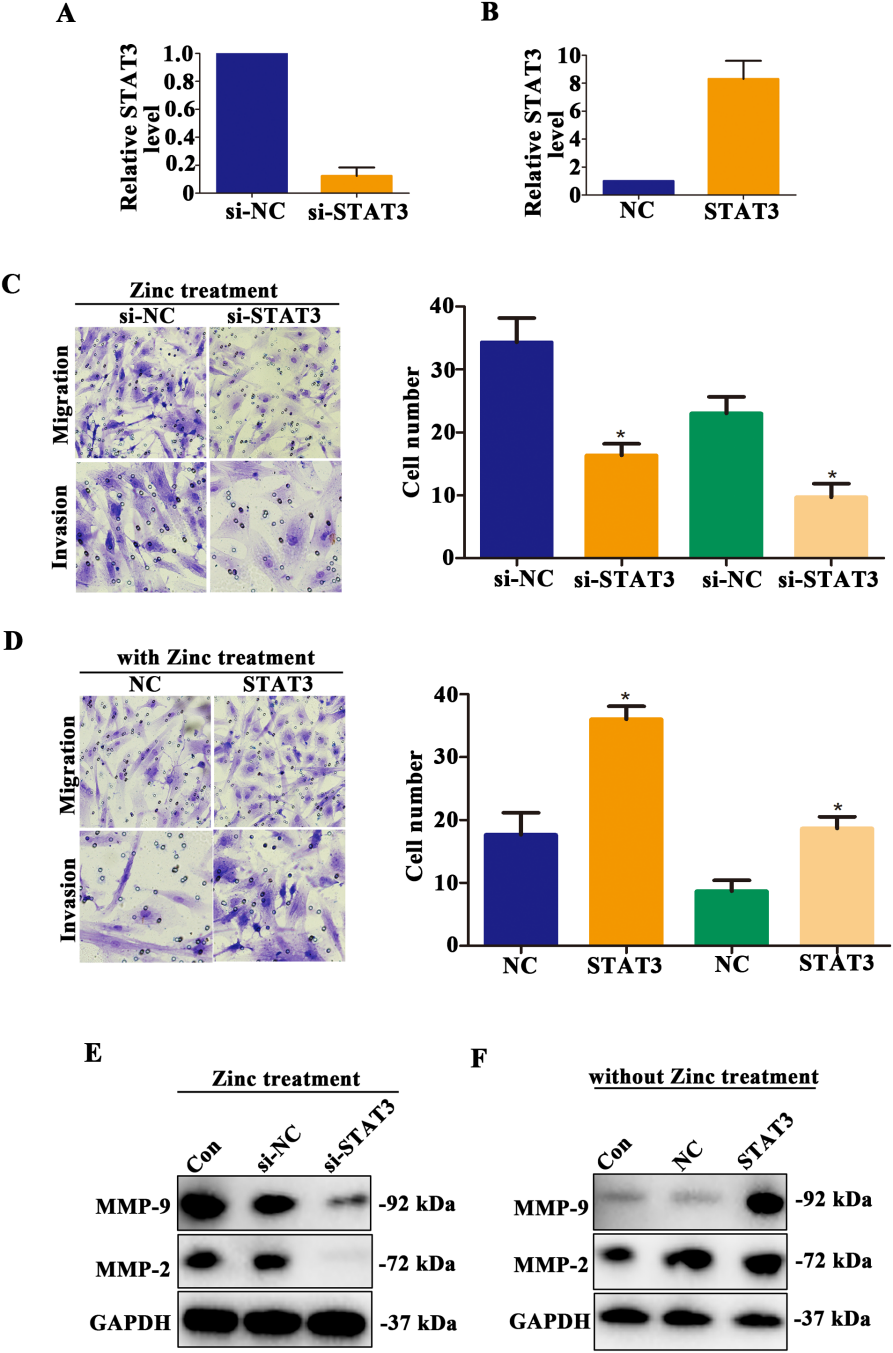
Zinc promotes HTR-8/SVneo cells migration and invasion mediated by STAT3 **(A-B)** The relative STAT3 mRNA level was detected by qRT-PCR. **(C)** After zinc treatment, Transwell assay was used to assess the abilities of migration and invasion in HTR-8/SVneo cells knocked down STAT3. **(D)** Transwell assay was used to assess the abilities of migration and invasion in HTR-8/SVneo cells overexpressed STAT3. **(E)** In zinc treatment group, the expression level of MMP-2 and MMP-9 was detected in HTR-8/SVneo cells knocked down STAT3. **(F)** In non-zinc treatment group, the expression level of MMP-2 and MMP-9 was detected in HTR-8/SVneo cells overexpressed STAT3.

Despite zinc treatment could promote trophoblast cell migration and invasion and up-regulate MMP-2/9 expression level, whether which mediated by STAT3 was unknown. HTR-8/SVneo cells of knockdown or overexpression of STAT3 were established, and the relative expression levels of STAT3 were detected by qRT-PCR (Figure 5A and 5B). Next, we found that knockdown the expression of STAT3 of HTR-8/SVneo cells after Zinc treatment, the abilities of migration and invasion were obviously decreased compared to those cells treated by Zinc solely (Figure 5C, *P* < 0.05). Contrary, when untreated HTR-8/SVneo were transfected with STAT3 expression plasmid, the abilities of migratory and invasive were increased compared to negative control cells (Figure 5D, *P* < 0.05). Similarly, knockdown the expression of STAT3 of HTR-8/SVneo cells after zinc treatment, the expression of MMP-2/9 were decreased compared to negative control cells (Figure 5E). Contrary, when untreated HTR-8/SVneo were transfected with STAT3 expression plasmid, the expression of MMP-2/9 were increased compared to negative control cells (Figure 5F). Taken together, it is STAT3-dependent that zinc promotes migration and invasion of HTR-8/SVneo cells via up-regulating MMP-2/9 expression.

## DISCUSSION

Our previous study showed that FGR can affect the regular development of fetal brain, and it can also damage the early and long-term spatial learning ability of neonates, plus the close association of FGR with the dysfunction of the central nervous system, leading to the abnormal behavior of FGR kids [3]. Recently, there is considerable evidence to support that learning and memory abilities impairment of FGR rats associated with placenta, especially for the deficiency of trophoblast cell invasion and migration [1]. In the present study we firstly showed that zinc supplement improved memory and learning abilities of FGR rats, and increased the abilities of invasion and migration of trophoblast cells. Moreover, the expression level of STAT3 was obviously up-regulated after zinc treatment, and promotes cell invasion and migration via increasing the expression level of MMP-2/9. We provide evidence that zinc improves learning and memory abilities of FGR rat via promoting trophoblast cell invasion mediated by STAT3-MMP-2/9 axis.

Now substantial evidence support that microelement have close relations with human health [16]. In the nervous system, zinc(Zn), vitamin D and selenium (Se) have been positively associated with neurodevelopment in early life [3, 17, 18]. Especially, prenatal zinc status was associated with child psychomotor abilities within the first year of life [19]. In clinical level, previous study found that prematurity lead to head growth restriction via influencing zinc levels [20]. Although more and more studies confirmed that zinc was related with neurodevelopment, study about whether zinc supplement could improve learning and memory abilities of FGR newborn was limit. *In vivo*, we firstly observed that zinc supplementation obviously improved brain weight and learning and memory abilities of FGR rats during pregnancy compared to non-Zinc treatment. Although this is the first report that zinc supplementation could reverse the impairment of learning and memory abilities abilities of FGR rats, the molecular mechanism was various. For example, zinc involves in regulating exchange of oxygen and nutrients [1], inflammatory reaction [21], vascular smooth muscle cells response [22], *etc*. Hence, it is necessary to further explore the potential mechanism of zinc on learning and memory abilities abilities of FGR rats.

Recently, accumulating evidence suggested that placental dysfunction underlies major obstetric diseases such as pre-eclampsia and FGR [23]. EVT, a subset of placental cells, plays a critical role in this process. EVT cells migrate and invade into the uterine wall, leading to remodeling of the maternal vasculature. However, inadequate invasion and migration abilities of EVT cells usually leading to FGR [1]. Recently, some novel molecular target was verified effective to promote EVT cells migration and invasion. For example, H19 long noncoding RNA alters trophoblast cell migration and invasion by regulating TβR3 in placentae with fetal growth restriction [1]. Transforming growth factor β1 promotes invasion of human JEG-3 trophoblast cells via TGF-β/Smad3 signaling pathway [6]. In present study, we firstly found that zinc treatment significantly promotes cell invasion and migration, which was associated with up-regulated STAT3 level. Previous study showed that upregulated MMP-2/9 contribute to cells migration and invasion abilities through STAT3-dependent signaling pathway in tumor cells [24]. Similarly, we also observed that zinc treatment could increase the expression of MMP-2/9, which was mediated by STAT3. Differently, we firstly verified the role of zinc supplement on invasion and migration in trophoblast cells, which may be a main cause of cognitive function improvement of FGR rats. Moreover, *in vivo*, we also observed the effect of zinc supplement on cognitive function improvement of FGR newborn rats. However, additional studies, including perform differential expression analysis of brain tissues from FGR rats by next generation sequencing or mRNA microarray are necessary. Perhaps additional novel evidence from brain tissue or new mechanisms would be found. In this study, we only focus on the effects of zinc on learning and memory in rats, but it is necessary to analyzed the relationship between zinc level and learning and memory abilities in clinical level in future. Moreover, it is unknown that whether zinc supplementation could also affect non-FGR rats in this study, but a previous randomized placebo controlled double blind clinical trial with healthy volunteers confirmed that zinc may reduce nitrosative stress and silent inflammation, and consequently the risk for various forms of degenerative diseases [25]. Hence, we should also pay more attention to the effect of zinc supplementation on learning and memory abilities in healthy population. Our findings represent the first example of zinc-based learning and memory abilities improvement of FGR rats and promotes trophoblast cell invasion and migration probably via enhancing STAT3-MMP-2/9 axis activity. Overall, zinc supplement was expected to be used for the treatment of learning and memory abilities impairment of FGR newborn.

## MATERIALS AND METHODS

### FGR animal model and zinc supplementation

A total of 58 healthy adult Sprague-Dawley (SD) rats (42 female and 16 male rats) weighing about 250 g were purchased from Beijing Vital River Laboratory Animal Technology Co., Ltd. Establishmen of pregnant model referred to our previous report [3]. Forty pregnant rats were randomly divided into the experiment group and the control group with 20 rats in each group. From the 2nd to 21st day, all the pregnant rats were placed into the smoking house 3 times for passive smoking (50 min/ time, and each time 4 cigarettes), respectively at 9:00 a.m., 12:00 a.m. and 4:00 p.m. Regular diet were taken for rats in the control group, and experimental group was fed with diet contain high levels zinc (0.2 μg, kg/day). The zinc levels in peripheral blood of all pregnant rats were detected by Zinc Assay Kit (Biovision Inc, Mountain View, CA). At different points in time, one was selected randomly from newborns of each pregnant rats to observe brain weight. This study was approved by the Animal Ethics Committee of Xi'an Jiaotong University.

### Morris water maze test

A self-made round pool (50 cm of diameter) was prepared, to which the water mixed with ink (25°C) was added. The pool was divided into 4 quadrants, and a refuge platform (6×6 cm) was set at 1 cm under the water in one of the quadrants and the position was fixed. According to with or without zinc supplementation, the rats were divided into experimental group and control group, Marris water maze test was performed according to previous report [3], and average scores were calculated.

### Passive avoidance test

A self-made transparent box (30×30×40 cm) was prepared, in which the steel tubes were fixated (2 cm in diameter) in parallel with the distance between every two tubes being 1 cm. Electric shock machine was installed on either end of each tube, and the insulated and safe platform (8×2.5×2.5 cm) was placed on the tubes in the corner at the bottom of the transparent box. After experiencing the Morris water maze test, passive avoidance test were performed in the two group rats according to previous report [3], and average scores were calculated.

### Microarray and bioinformatic analyses

The placental tissues were isolated from pregnant rats with or without zinc treatment, and gene expression array was performed by the Affymetrix GeneChip® HT RG-230 PM Array Plate (Shanghai Bohao Biotech Co., Ltd., Shanghai, China). Gene Ontology (GO) and Kyoto Encyclopedia of Genes and Genomes (KEGG) were used to analyze the function and pathways of these differentially expressed mRNAs. GO and KEGG analyses were conducted using the String online analysis tool (https://string-db.org/). String database used to analyzed the protein-protein interaction(PPI) among main differential genes(https://string-db.org/).

### Cell culture and transfection

HTR-8/SVneo cells were preserved in our laboratory and were cultured in RPMI1640 (Gibco, Invitrogen, NY, USA) supplemented with 10% fetal bovine serumb(Gibco) and 1% penicillin/streptomycin(Sigma-Aldrich, St Louis, MO, USA), and cultivated in an incubator at 37°C with 5% CO_2_. Zinc solution was purchased from Shanghai Jingdu Biotechnology Co. Ltd.(1000μg/mL, Shanghai, China). In experiment group, Zinc solution (10μM) [26] was used to treat HTR-8/SVneo. After 48 hours, these cells were used to for subsequent experiments. pcDNA 3.0-STAT3 and STAT3 sh-RNA plasmids were preserved in our laboratory. Cells were transfected with 2.5 μg plasmid using Lipofectamine 2000(Invitrogen, Carlsbad, CA, USA), according to the manufacturer’s instructions.

### Western blot analysis

Cells were collected and lysed in radioimmunoprecipitation buffer (Beijing CoWin Biotech Co., Ltd., Beijing, China) with protease inhibitors for 30 min to extract total proteins. BCA Protein Assay Kit (Thermo Fisher Scientific, Waltham, MA, USA) was used to quantity protein level. 40 ug total protein were loaded onto 10% SDS gel, followed by Western blot analysis. Then transferred to membrane, and incubated overnight at 4°C with primary antibodies targeting anti-STAT3(abcam, Cambridge, MA, USA, dilution 1:1000), anti-STAT3 (phospho Y705)(abcam, dillution 1:50000), anti-MMP-2(abcam, dillution 1:500) and anti-MMP-9(abcam, dillution 1:1000), anti-GAPDH (Beijing Zhongshan Golden Bridge Biotechnology Co. Ltd., Beijing, China; dillution 1:1000), were followed by incubation with an HRP-conjugated goat anti-rabbit secondary antibody (Beijing Zhongshan Golden Bridge Biotechnology Co. Ltd.; dillution 1:1000) for 1 h at room temperature. Immunocomplexes were detected using an enhanced chemiluminescence kit (Thermo Fischer Scientific), and images were analyzed using Image J software (version 1.62; National Institute of Health, Bethesda, MD, USA).

### RNA isolation and real time PCR

Total RNAs were isolated from HTR-8/SVneo cells using Trizol reagent (Invitrogen) and was reverse-transcribed into cDNA using the ReverTra Ace kit (Toyobo, Osaka, Japan) according to the manufacturer’s instruction. Real-time quantitative PCR was carried out in a 20 μl reaction containing 0.6 μl of cDNA using the ABI7500 real-time PCR system(Applied Biosystems, Foster City, CA, USA). GAPDH was used as an internal control in each sample.

### Transwell assay

For migration assay, Transwell chambers (Corning, 8μm pores) placed into a 24-well plate (Thermo Fisher Scientific) were used in the assays. The lower chamber was filled with 600 μl RPMI 1640 containing 10% FBS. And 5 × 10^4^ cells in 100 μl serum-free RPMI 1640 were added to the upper chamber. The cells were allowed to migrate for 20 h at 37°C before fixing. For invasion assays, Matrigel (BD Biosciences, Bedford, MA, USA) was pre laid on the membrane of upper chamber, the others was same as above. Cells passed through membrane were counted under microsopy, and the average of three independent experiments were calculated.

### Statistical analysis

SPSS 21.0 software (Chicago, IL, USA) was used for processing the data. Measurement data were presented as mean ± SD. Data from various experiments were analyzed by independent-samples t-testing. *P*<0.05 indicated that the difference was statistically significant.
